# Clinical characteristics and risk factors of chronic critical illness in children with sepsis

**DOI:** 10.3389/fped.2025.1561044

**Published:** 2025-05-02

**Authors:** Lin Yang, Na Zang, Cong Liu, Ying Yang, Kai Bing Pu, Li Ping Tan, En Mei Liu

**Affiliations:** ^1^ Department of Emergency, Children’s Hospital of Chongqing Medical University, Chongqing, China; ^2^ National Clinical Research Center for Child Health and Disorders, Ministry of Education Key Laboratory of Child Development and Disorders, Chongqing Key Laboratory of Child Rare Diseases in Infection and Immunity, Chongqing, China; ^3^ Department of Respiratory, Children’s Hospital of Chongqing Medical University, Chongqing, China; ^4^ Department of Infection, Children’s Hospital of Chongqing Medical University, Chongqing, China

**Keywords:** pediatric sepsis, chronic critical illness (CCI), clinical characteristics, risk factors, immunosuppression

## Abstract

**Objectives:**

This study aimed to examine the clinical characteristics and risk factors associated with the development of chronic critical illness (CCI) in children with sepsis.

**Methods:**

A retrospective analysis was conducted on children diagnosed with sepsis and admitted to the Pediatric Intensive Care Unit (PICU) at Chongqing Medical University Affiliated Children's Hospital between January 2015 and December 2022. Patients were categorized into two groups based on clinical outcomes: CCI group, defined by an ICU stay ≥14 days with persistent organ dysfunction, and non-CCI group, including patients with rapid recovery or early death. Data on baseline demographics, clinical characteristics, and diagnostic and therapeutic differences were collected and analyzed.

**Results:**

Among 1,326 children with sepsis, 244 were classified in the CCI group (135 males, 109 females) and 1,082 were classified in the non-CCI group (651 males, 431 females), including 163 cases in the early death group and 919 cases in the rapid recovery group. No significant differences were observed between the groups in terms of sex, age distribution, or prevalence of septic shock. Respiratory and gastrointestinal infections were the predominant sources of infection in both groups. Compared to the non-CCI group, the CCI group exhibited significantly higher weights, pediatric sequential organ failure assessment (pSOFA) scores, rates of underlying respiratory diseases, trauma, surgical interventions, mechanical ventilation duration, ICU stay, total hospital stay, and secondary infection rates. Multivariate logistic regression identified pSOFA score, underlying respiratory diseases, trauma, prolonged mechanical ventilation, surgical interventions, and secondary infections as independent risk factors for the development of CCI in children with sepsis. Based on ROC analysis, the AUC of the established CCI prediction model was 0.902 (95% CI: 0.873–0.928). Secondary infections were also a prominent clinical feature of CCI cases.

**Conclusions:**

CCI in pediatric sepsis is associated with underlying respiratory diseases, trauma, elevated pSOFA scores, surgical procedures, prolonged mechanical ventilation and secondary infections. These factors contribute to extended hospital stays, elevated secondary infection rates, and poor clinical outcomes. The persistence of pro-inflammatory mediators and subsequent immunosuppression may underlie the development of CCI in this population.

## Background

Sepsis is a complex clinical syndrome resulting from a dysregulated host response to infection, leading to life-threatening organ dysfunction and posing a significant threat to pediatric health. Approximately 25 million children are affected by sepsis each year, resulting in over 3 million deaths (1). In resource-limited regions, particularly in Africa and Asia, sepsis is the leading cause of mortality among children under the age of 5, accounting for 36.7% of all childhood deaths (2). While significant advancements in diagnostic and therapeutic strategies have reduced in-hospital mortality rates, with age-standardized sepsis mortality declining by 52.8% (47.7%–57.5%) between 1990 and 2017 (1), this reduction has not translated to improved long-term outcomes. Rather, increased short-term survival among sepsis patients has been linked to an increased incidence of chronic critical illness (CCI), with up to 33% of survivors developing this condition (3, 4). Patients with CCI frequently experience late complications of sepsis, which significantly contribute to mortality. However, much of the current understanding of CCI stems from studies on adult populations, leaving substantial gaps in the epidemiological and clinical characterization of CCI in children following sepsis.

CCI refers to patients who survive the initial hyperinflammatory phase of sepsis, commonly referred to as the “cytokine storm”, but fail to achieve full recovery, requiring prolonged critical care. Although a universally accepted definition is lacking, CCI is broadly characterized by persistent organ dysfunction necessitating extended hospitalization and substantial resource utilization, including prolonged intensive care unit (ICU) stays (5, 6). A 2-week ICU stay, as defined by length-of-stay data from Shands UF Health, serves as the standard for identifying CCI (7). Evidence suggests that patients with CCI experience higher rates of secondary infections, longer hospital stays, and poorer outcomes (8). Approximately 60% of CCI patients are readmitted within six months (9), predominantly due to recurrent infections, with mortality rates reaching 40% within this period (10). Alarmingly, 70% of CCI patients succumb to their illness before the cessation of treatment (11). These findings underscore the severe burden of CCI and its significant implications for clinical management and outcomes.

CCI is partially attributed to a malfunctioning host response to prolonged activation of pattern recognition receptors (PRRs), leading to deregulated hematopoiesis, persistent inflammation, T cell decline, and an increase in immunosuppressive cell activities. These attributes hint that CCI might constitute a unique pathophysiological state with distinct mechanisms (12). The core pathology of sepsis encompasses immune dysregulation, with studies in adults revealing that CCI patients frequently enter a prolonged immunosuppressive phase, marked by heightened vulnerability to secondary infections and elevated mortality risks. Although there's a prevalent clinical belief linking CCI to chronic immunosuppression, solid clinical evidence supporting this link is still scant. Currently, the underlying mechanisms of immunosuppression and inflammation in sepsis remain elusive, hindering efforts to prevent secondary infections and enhance long-term outcomes. To bridge these knowledge gaps, this study compared the clinical profiles of pediatric sepsis patients with prolonged vs. swift recovery, assessed risk factors for CCI development, and delved into the biological and pathophysiological features and mechanisms underlying the diverse clinical courses.

## Subjects and methods

### Study population

This retrospective observational study analyzed 1,353 pediatric sepsis patients admitted to the Pediatric Intensive Care Unit (PICU) of Chongqing Medical University Affiliated Children's Hospital between January 2015 and December 2022. Comprehensive data were collected for each patient, including demographic characteristics, clinical manifestations, underlying diseases, laboratory test results, secondary infections, treatment interventions, and final outcomes.

### Inclusion and exclusion criteria

The inclusion criteria for the study included: (1) Patients admitted to the ICU; (2) Age range 0–18 years; (3) Clinically diagnosed with sepsis or septic shock, representing the patient's first episode of sepsis; and (4) Patients enrolled in the sepsis clinical management protocol. The exclusion criteria for the study included: (1) Patients not admitted to the PICU or not meeting the diagnostic criteria for sepsis; (2) Severe traumatic brain injury, evidenced by a Glasgow Coma Scale (GCS) score <8 and confirmed neurological injury on CT imaging; and (3) Patients with incomplete medical records.

Approval for conducting the study was granted by the Ethics Committee of Chongqing Medical University Affiliated Children's Hospital (Approval Number: 534/2022). Guardians of all participants were informed and provided their consent, adhering to ethical guidelines for research.

## Data collection and methods

### Data collection

Clinical data were obtained retrospectively through the hospital's electronic medical record system and supplemented by follow-up visits. Demographic variables included sex, age, and weight. Clinical characteristics included the primary source of infection, severity of sepsis (whether complicated by septic shock), organ dysfunction scores, and presence of underlying diseases. Laboratory tests encompassed essential indicators like white blood cell count, neutrophil count, lymphocyte count, hemoglobin levels, platelet count, procalcitonin, bilirubin, creatinine, albumin, and D-dimer levels. The treatment-related data documented the use of vasoactive medications, cardiopulmonary resuscitation, the duration of mechanical ventilation, surgical interventions, blood transfusions, blood purification therapies, length of ICU stay, and overall hospital stay. Additionally, secondary infection records were reviewed, noting the occurrence and specific anatomical sites of infections acquired during hospitalization. These comprehensive datasets provided a robust basis for subsequent analysis.

### Diagnostic criteria

The diagnosis of sepsis was established using the pediatric sequential organ failure assessment (pSOFA) score, which assesses dysfunction across various organ systems, including respiratory, coagulation, liver, and kidney functions. A pSOFA score of 2 or higher was deemed indicative of sepsis, in line with established guidelines (13). Septic shock was characterized as severe infection leading to cardiovascular dysfunction, manifested by hypotension, the need for vasoactive drug treatment, or signs of perfusion abnormalities. Additionally, the “2024 International Consensus Standards for Pediatric Sepsis and Septic Shock” were adopted, incorporating the Phoenix Sepsis Score (PSS) for a comprehensive evaluation. The PSS assesses cardiovascular, respiratory, neurological, and coagulation functions, with a score of 2 or higher confirming sepsis in children with suspected or confirmed infections. Further, septic shock was defined as sepsis accompanied by a PSS cardiovascular score of at least 1 (14).

### Group classification

Pediatric patients diagnosed with sepsis or septic shock in our hospital's PICU were categorized into three groups based on their clinical courses: rapid acute progression (RAP, comprising 919 cases), early death (163 cases), and complex chronic immunosuppression (CCI, 244 cases). The CCI group was distinguished by clinical immunosuppression, often manifested through secondary infections following sepsis.

The CCI group encompassed patients with an ICU stay of at least 14 days and persistent organ dysfunction, as determined by a cardiovascular pSOFA score of ≥1 or any other organ system pSOFA score of ≥2 at day 14. Additionally, patients with an ICU stay of less than 14 days who were transferred to another hospital, a long-term acute care facility, or hospice while still exhibiting organ dysfunction at discharge were also included in the CCI group. Patients who did not meet the CCI criteria were placed in the non-CCI group, which encompassed those in the rapid recovery and early death categories.

### Statistical analysis

The data were analyzed using SPSS version 26.0 statistical software. Categorical variables were presented as frequencies (*n*) and percentages (%), with group differences tested using the Chi-square (*χ*^2^) test. Non-normally distributed data were described using medians with interquartile ranges (Q1, Q3), and group comparisons were conducted using the Mann–Whitney *U* test. To identify factors linked to study outcomes, univariate analysis was initially used to screen potential risk factors. Subsequently, Lasso regression was applied to select more robust predictive variables, retaining only those with statistical significance (*P* < 0.05). Multivariate logistic regression analysis, utilizing the backward elimination method, was employed to quantify the risks associated with the selected predictive variables. The predictive performance of the final model was assessed using the area under the receiver operating characteristic curve (AUROC). Statistical significance was established at a two-tailed *P* < 0.05.

## Results

### Patient demographics and sepsis characteristics

This study included 1,326 pediatric sepsis patients, comprising 244 cases in the CCI group (135 males and 109 females), including 28 cases in the CCI mortality group and 216 in the survival group. The remaining 1,082 cases were classified in the non-CCI group (651 males and 431 females), including 163 cases in the early death group and 919 in the rapid recovery group. The CCI group accounted for 18.4% of pediatric sepsis patients, with a mortality rate of 11.5%, both significantly lower than the proportions found in adult studies (3). In contrast, the rapid recovery group accounted for 69.3% of the cohort, indicating a generally favorable prognosis for the majority of pediatric sepsis patients. Children in the CCI group were older and had higher body weights compared to those in the non-CCI group. Additionally, both hospital and PICU stays were significantly longer for patients in the CCI group (*P* < 0.05, Table 1).

**Table 1:** Comparison of patient demographics between the two groups.

Demographics		Entire cohort (1,326)	Non CCI (1,082)	CCI (244)	*P* value
Male, *n* (%)		786 (59.3%)	651 (60.2%)	135 (55.3%)	0.997
Age in years, median (25th, 75th)		1.42 (0.33, 5.00)	1.33 (0.33, 5.00)	1.75 (0.50, 7.25)	0.023
Age distribution	28 day–1 year	589 (44.4%)	495 (45.7%)	94 (38.5%)	/
	3 year	325 (24.5%)	264 (24.4%)	61 (25.0%)	
	9 year	250 (18.9%)	200 (18.5%)	50 (20.5%)	
	14 year	145 (10.9%)	110 (10.2%)	35 (14.3%)	
	18 year	17 (1.3%)	13 (1.20%)	4 (1.64%)	
Weight median (25th, 75th) (kg)		10.00 (6.00, 18.00)	10.0 (6.00, 17.00)	11.2 (6.50, 22.00)	0.015
ICU LOS, median (25th, 75th)		6.00 (3.00, 11.00)	5.00 (2.00, 8.00)	20.0 (16.00, 27.00)	<0.001
Hospital LOS, median (25th, 75th)		17.50 (10.00, 30.00)	15.0 (8.00, 24.00)	35.0 (25.00, 46.00)	<0.001

### Clinical characteristics of CCI

In the CCI group, respiratory infections were the most common source of sepsis, followed by gastrointestinal infections, with the overall distribution of infection sources resembling that of the control group. Slight differences were observed in the prevalence of other sources of infection between the groups. Regarding sepsis severity, the incidence of septic shock was comparable between the CCI and control groups. However, patients in the CCI group exhibited significantly higher pSOFA scores across most parameters, except for liver function, as well as higher total pSOFA scores (*P* < 0.05, Table 2). These findings suggest a strong association between the degree of organ dysfunction and both disease outcome and the development of CCI.

**Table 2:** Comparison of characteristics between the two groups.

Sepsis characteristics		Entire cohort (1,326)	Non CCI (1,082)	CCI (244)	*P* value
Source of sepsis infection, *n* (%)	Intra-abdominal sepsis	210 (15.8%)	168 (15.5%)	42 (17.2%)	0.579
	Pneumonia	899 (67.8%)	723 (66.8%)	176 (72.1%)	0.127
	NSTI	79 (6.0%)	74 (6.84%)	5 (2.05%)	0.007
	Bacteremia	109 (8.2%)	102 (9.43%)	7 (2.87%)	0.001
	Surgical site infection	4 (0.3%)	1 (0.09%)	3 (1.23%)	0.021
	Urosepsis	14 (1.1%)	13 (1.20%)	1 (0.41%)	0.487
	CLABSI	15 (1.1%)	10 (0.92%)	5 (2.05%)	0.171
	Other	11 (0.8%)	8 (0.74%)	3 (1.23%)	0.435
Sepsis severity, *n* (%)	Septic shock	651 (49.1%)	527 (48.7%)	124 (50.8%)	0.599
Pediatric SOFA score	SOFA score	5 (3, 8)	5 (3, 8)	8 (4. 10)	<0.001
	Neurological	0 (0, 1)	0 (0, 1)	1 (0, 2)	<0.001
	Hepatic	0 (0, 0)	0 (0, 0)	0 (0, 0)	0.147
	Renal	0 (0, 1)	0 (0, 1)	0 (0, 0)	<0.001
	Coagulation	0 (0, 2)	0 (0, 1)	1 (0, 2)	<0.001
	Cardiovascular	1 (0, 3)	1 (0, 2)	2 (0, 3)	0.004
	Respiratory	3 (1, 3)	3 (0, 3)	3 (3, 3)	<0.001

### Analysis of underlying diseases

The proportion of patients with underlying diseases in the CCI group was significantly higher than that in the control group. Notably, patients with respiratory system disorders, such as bronchopulmonary dysplasia and congenital airway and lung maldevelopment, as well as those with trauma, demonstrated a significantly higher likelihood of progressing to CCI (*P* < 0.05, Table 3).

**Table 3:** Comparison of underlying diseases between the two groups.

Underlying disease	Entire cohort (1,326)	Non CCI (1,082)	CCI (244)	*P* value
Cardiovascular diseases	203 (15.3%)	166 (15.3%)	37 (15.2%)	1.000
Immune system diseases	79 (6.0%)	67 (6.19%)	12 (4.92%)	0.542
Hematological system diseases	109 (8.2%)	92 (8.50%)	17 (6.97%)	0.509
Other tumor-related diseases	46 (3.5%)	36 (3.33%)	10 (4.10%)	0.688
Genetic metabolic diseases	209 (15.8%)	161 (14.9%)	48 (19.7%)	0.079
Respiratory system diseases	27 (2.0%)	17 (1.57%)	10 (4.10%)	0.020
Connective tissue diseases	34 (2.6%)	23 (2.13%)	11 (4.51%)	0.057
Neurological disorders	110 (8.3%)	96 (8.87%)	14 (5.74%)	0.140
Hepatic failure	13 (1.0%)	12 (1.11%)	1 (0.41%)	0.482
Renal failure	15 (1.1%)	14 (1.29%)	1 (0.41%)	0.330
After transplantation surgery	22 (1.7%)	19 (1.76%)	3 (1.23%)	0.782
After cardiopulmonary resuscitation	38 (2.9%)	33 (3.05%)	5 (2.05%)	0.526
Trauma	19 (1.4%)	8 (0.74%)	11 (4.51%)	<0.001
Drowning, feces drowning	6 (0.5%)	3 (0.28%)	3 (1.23%)	0.080
Merge ≥2 types	211 (15.9%)	175 (16.2%)	36 (14.8%)	0.652
Nothing	608 (45.9%)	513 (47.4%)	95 (38.9%)	0.020

### Laboratory parameters

No significant differences were observed in white blood cell count (WBC), hemoglobin (Hb) levels, absolute lymphocyte count, absolute neutrophil count, platelet count, bilirubin levels, creatinine levels, or albumin levels between the CCI and control groups. However, procalcitonin (PCT) was significantly lower, while D-dimer levels were significantly higher in the CCI group compared to the control group (*P* < 0.05, Table 4).

**Table 4:** Comparison of laboratory indicators between the two groups.

Characteristics	Entire cohort (1,326)	Non CCI (1,082)	CCI (244)	*P* value
WBC	10.34 (5.67, 16.64)	10.1 (5.66, 16.1)	11.6 (5.92, 17.7)	0.076
Hb	100 (88, 113)	100 (87.2, 113)	100 (88.0, 115)	0.834
Lymphocyte count	2.33 (1.18, 4.49)	2.31 (1.18, 4.51)	2.45 (1.27, 4.37)	0.692
Neutrophil count	6.52 (2.99, 11.83)	6.43 (2.92, 11.5)	6.98 (3.08, 12.4)	0.207
Platelet count	257.5 (114, 414)	259 (112, 416)	242 (116, 406)	0.991
PCT	4.47 (0.87, 17.59)	4.80 (1.12, 16.9)	2.62 (0.36, 21.2)	0.043
Bilirubin	6.60 (3.10, 14.70)	6.70 (3.10, 15.0)	6.20 (3.38, 13.2)	0.801
Creatinine	32.05 (22.60, 54.00)	32.1 (22.5, 54.6)	32.0 (23.0, 52.8)	0.928
Albumin	31.35 (25.50, 37.90)	31.6 (25.3, 38.1)	31.0 (26.0, 37.0)	0.883
D-dimer	2.93 (1.16, 8.60)	2.77 (1.11, 8.03)	4.03 (1.51, 10.3)	0.003

### Treatment and secondary infections

The CCI group demonstrated a significantly greater reliance on vasoactive drugs, along with significantly higher rates and durations of mechanical ventilation use, frequencies of surgical procedures, and use of blood purification therapies compared to the control group (*P* < 0.05, Table 5).

**Table 5:** Comparison of treatment between the two groups.

Treatment measure	Entire cohort (1,326)	Non CCI (1,082)	CCI (244)	*P* value
Vasoactive drugs (*n*)	584 (44.0%)	459 (42.4%)	125 (51.2%)	0.015
Cardiopulmonary resuscitation (*n*)	128 (9.7%)	108 (9.98%)	20 (8.20%)	0.464
Mechanical ventilation (*n*)	996 (75.1%)	765 (70.7%)	231 (94.7%)	<0.001
Days of mechanical ventilation	2.00 (0.20, 6.00)	1.00 (0.00, 4.00)	11.0 (5.00, 16.2)	<0.001
Surgical procedure (*n*)	314 (23.7%)	236 (21.8%)	78 (32.0%)	0.001
Blood transfusion (*n*)	443 (33.4%)	375 (34.7%)	68 (27.9%)	0.050
Chemotherapy (*n*)	65 (4.9%)	51 (4.71%)	14 (5.74%)	0.613
Blood purification (*n*)	178 (13.4%)	122 (11.3%)	56 (23.0%)	<0.001
Number of secondary infections, *n* (%)	495 (37.3%)	353 (32.6%)	142 (58.2%)	<0.001

Secondary infections were more prevalent and numerous in the CCI group compared to the control group, with significantly higher rates of respiratory, urinary tract, surgical site, bloodstream, catheter-related, and fungal infections. These differences suggest a strong association between secondary infections and the progression of sepsis into CCI. Furthermore, the high incidence of secondary infections highlights their role as a key clinical characteristic of CCI, reflecting a greater degree of immunosuppression in this group.

### Risk factors and diagnostic value analysis

Univariate analysis was conducted to preliminarily screen potential risk factors, retaining those variables with statistical significance (*P* < 0.05). These variables were further refined using a Lasso regression model to identify robust predictors. Subsequently, multivariate logistic regression analysis was performed to assess the risk factors for CCI in pediatric sepsis patients (*P* < 0.05, Table 6). Multivariate logistic regression analysis identified pSOFA score, underlying diseases, trauma, days of mechanical ventilation, and surgical procedures as independent risk factors for the development of CCI, with necrotizing soft tissue infection (NSTI) and blood transfusions identified as potential protective factors (*P* < 0.05, Table 7). Additionally, secondary infections were found to function as both a significant risk factor and a defining clinical characteristic of CCI.

**Table 6:** Multivariate regression analysis of CCI patients.

Clinical variables	OR	95% CI	*P* value
pSOFA score	1.061	1.000–1.125	0.049
Source of sepsis infection (NSTI)	0.199	0.062–0.637	0.007
Underlying diseases (RSD)	3.27	1.204–8.882	0.02
Underlying diseases (trauma)	8.489	2.378–30.307	<0.001
Days of mechanical ventilation	1.451	1.381–1.524	<0.001
Surgical procedures	2.186	1.402–3.409	<0.001
Blood transfusions	0.541	0.346–0.846	0.007
Secondary infections	2.471	1.640–3.723	<0.001

**Table 7:** ROC curve analysis of independent predictors of CCI patients.

Clinical variables	AUC	95% CI
pSOFA score	0.663	0.629–0.698
Source of sepsis infection (NSTI)	0.524	0.485–0.563
Underlying diseases (RSD)	0.513	0.472–0.553
Underlying diseases (trauma)	0.519	0.478–0.560
Days of mechanical ventilation	0.869	0.839–0.899
Surgical procedure	0.551	0.510–0.592
Blood transfusion	0.534	0.495–0.573
Secondary infections	0.628	0.588–0.667
Classifier	0.902	0.876–0.928

**Figure 1:**
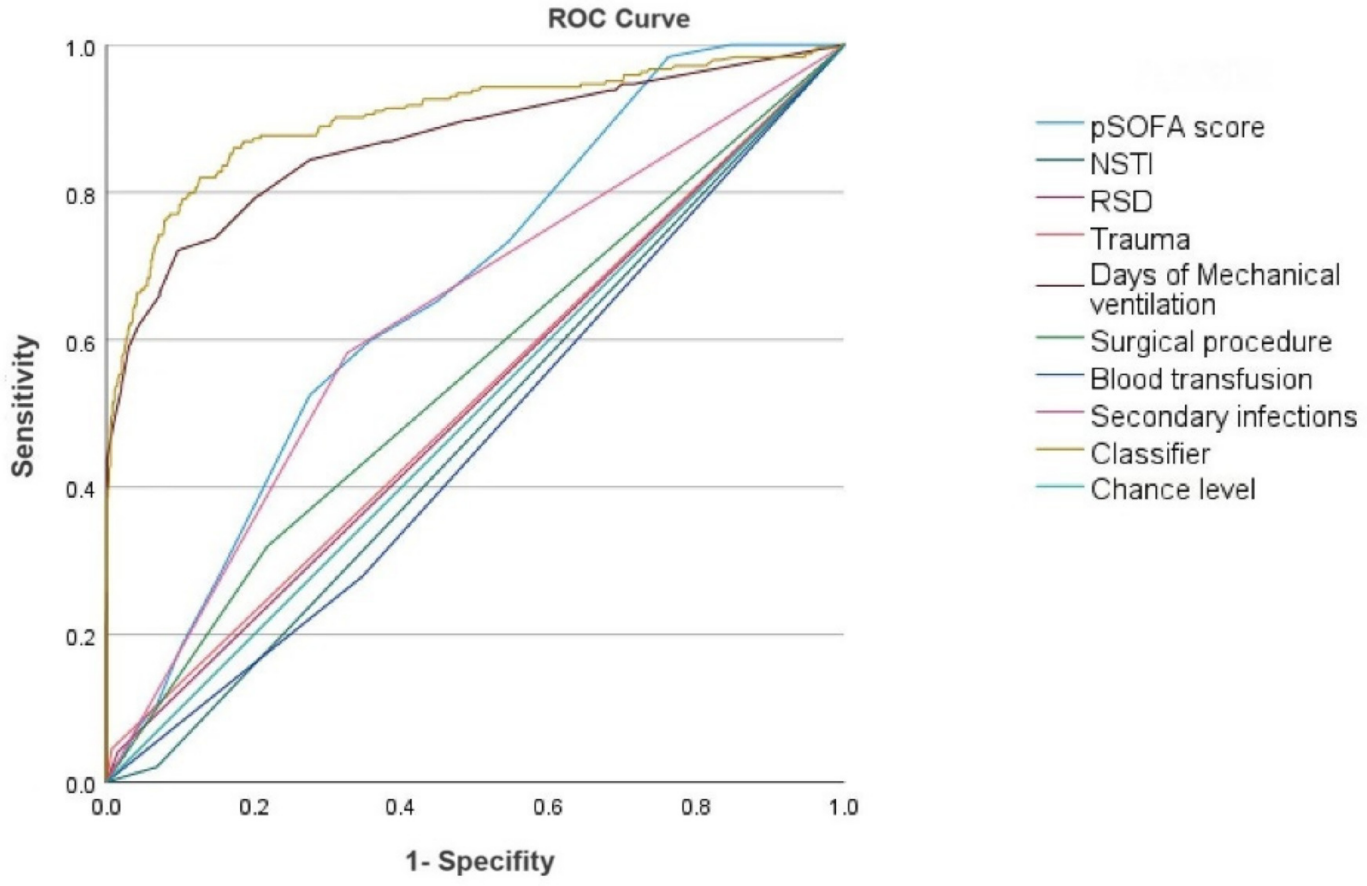
ROC curve analysis of independent predictors of CCI in children with sepsis. AUC: 0.902 (0.876–0.928).

ROC curves were plotted for pSOFA score, source of sepsis infection (NSTI), underlying diseases, trauma, days of mechanical ventilation, surgical procedures, blood transfusion, and secondary infections (Figure 1), with AUC values of 0.663, 0.524, 0.513, 0.519, 0.869, 0.551, 0.534, and 0.628, respectively. The ROC curve for days of mechanical ventilation had the largest AUC, indicating the greatest predictive value for sepsis leading to CCI. Based on ROC analysis, the AUC of the established CCI prediction model was 0.902 (95% CI: 0.873–0.928).

## Discussion

CCI following sepsis is an increasingly prominent issue in critical care medicine. Although standardized diagnostic criteria are lacking, CCI is widely defined as persistent organ dysfunction requiring an ICU stay exceeding 14 days, reflecting a transition from acute organ dysfunction to chronic failure with ongoing organ dysfunction (10, 15). Recent studies have highlighted several risk factors for the development of CCI following sepsis, including advanced age, male sex, and pre-existing conditions (16, 17). A key contributor to the progression of CCI is sepsis-induced immunosuppression, which markedly increases the risk of secondary infections (15, 18). This association underscores the critical interplay between immune dysregulation and the pathogenesis of CCI, with secondary infections not only complicating clinical management but also exacerbating long-term outcomes. Despite these insights, research on pediatric populations remains notably limited. Data on epidemiology, clinical characteristics, and mechanisms underlying CCI in children following sepsis are scarce, and the absence of pediatric-specific diagnostic criteria further complicates efforts to address this condition. This knowledge gap is a considerable obstacle to formulating effective prevention and treatment strategies. This study identified key independent risk factors for the development of CCI by comparing patients in the RAP and early death groups. These factors included higher pSOFA scores, underlying respiratory diseases, trauma, prolonged mechanical ventilation, surgical interventions, and secondary infections.

This study was conducted at the largest pediatric intensive care unit (PICU) in Southwest China, involving a retrospective and in-depth analysis of clinical outcomes in children with sepsis over nearly eight years. Analysis revealed that 12.3% of patients experienced early mortality, while 18.4% progressed to CCI. The remaining 69.3% were categorized into an early recovery group, demonstrating favorable prognoses. The proportion of children developing CCI (18.4%) was lower than that reported in adults (33%) (3), although it remains clinically significant. Notably, the mortality rate in the pediatric CCI group (11.5%) was also significantly lower than that observed in adults, potentially attributable to the unique physiological characteristics and enhanced immune recovery capacity of children, combined with the advanced treatment capabilities of our center.

Both patient groups were similar in age, weight, sepsis severity, and infection sources, primarily respiratory infections. Our findings further highlighted a strong correlation between higher pSOFA scores and the development of CCI, indicating that severe organ dysfunction plays a crucial role in both sepsis prognosis and CCI prediction. Additionally, underlying medical conditions emerged as a significant factor influencing patient outcomes. Our study showed a notable link between these conditions and the progression of sepsis to CCI, potentially due to the immune and physiological disturbances they cause. Notably, patients with pre-existing conditions, particularly respiratory diseases like bronchopulmonary dysplasia and congenital airway or lung anomalies, faced a substantially greater risk of CCI. Conversely, those without such conditions had a relatively lower risk. This discrepancy could be attributed to the increased likelihood and extended duration of mechanical ventilation in patients with respiratory comorbidities, both of which contribute to CCI development. Trauma was also identified as a contributing factor to CCI, potentially due to the exacerbated inflammatory response, local hemodynamic disturbances, and heightened physiological stress triggered by injury. These findings underscore the multifactorial nature of CCI development and highlight the importance of addressing underlying and contributing factors to mitigate its progression.

Regarding laboratory parameters, there were no notable differences between the CCI and control groups in terms of WBC count, Hb levels, absolute lymphocyte count, absolute neutrophil count, platelet count, bilirubin levels, creatinine levels, or albumin levels. However, the CCI group exhibited significantly lower PCT levels and notably higher D-dimer levels compared to the control group. D-dimer is a specific biomarker generated during fibrinolysis and fibrin degradation following thrombosis, playing a critical role in the diagnosis and assessment of sepsis (19, 20). These observations align with the findings of the recently published Pediatric Sepsis Phoenix Sepsis Score (PSS) study by Schlapbach et al. (14). The potential mechanisms underlying elevated D-dimer levels, such as hypercoagulability, hyperfibrinolysis, or endothelial injury, suggest that increased D-dimer is associated with adverse clinical outcomes (21). This study underscores the importance of D-dimer as both a diagnostic and prognostic indicator in sepsis, with its abnormal elevations closely linked to disease severity and the risk of CCI in sepsis patients. Procalcitonin (PCT), as a relatively specific inflammatory marker for sepsis, plays a significant role in assessing disease severity (22). The host response varies depending on the type of pathogen, leading to varying degrees of PCT elevation. Since the biomarkers we compared were based on laboratory results obtained within 24 h of admission to the pediatric intensive care unit (PICU), our findings suggest that early PCT levels are not directly associated with the development of chronic critical illness (CCI) following sepsis. In future studies, we aim to further explore the relationship between these biomarkers and the pathophysiology of CCI to elucidate the underlying clinical phenomena.

In terms of treatment, patients in the CCI group required substantially longer durations of mechanical ventilation and more frequent vasoactive drug therapy, surgical interventions, and blood purification procedures compared to those in the RAP group. Univariate analysis identified key factors such as prolonged mechanical ventilation and surgical interventions as significant contributors to CCI development. In addition to trauma, patients undergoing surgical procedures demonstrated a higher risk of progressing to CCI. This is consistent with findings from surgical ICU populations, where over one-third of patients with abdominal sepsis develop CCI, often accompanied by poor long-term prognoses (16). These observations underscore the heightened vulnerability of surgical ICU survivors to prolonged critical illness and pronounced immunosuppression, emphasizing the need for targeted management strategies to mitigate these risks. In this study, intergroup analysis revealed a relatively higher transfusion rate in the pediatric sepsis chronic critical illness (CCI) group. The primary reasons for this observation include prolonged ICU stays, recurrent infections, and frequent blood sampling, all of which contribute to an increased risk of anemia and, consequently, a higher likelihood of transfusion. Therefore, transfusion should not be considered a true protective factor but rather a consequence of chronic critical illness.

Gentile et al. identified and characterized the clinical phenotype of CCI as persistent inflammation-immunosuppression and catabolism syndrome (PICS) (4). This syndrome, a hallmark of various conditions, including sepsis, trauma, advanced malignancies, and chronic inflammatory diseases, provides a novel framework for understanding the complex pathophysiological mechanisms underlying CCI. PICS highlights the interplay between persistent inflammatory responses and profound immunosuppression as central drivers of CCI progression. This is consistent with our findings, which identified immune dysregulation as a pivotal factor in the occurrence and development of CCI. Immune imbalance, a defining feature of sepsis pathogenesis, manifests in CCI patients as a prolonged state of immunosuppression, substantially increasing their susceptibility to secondary infections and mortality. In the current study, immunosuppression was clinically determined by the occurrence of post-sepsis secondary infections, consistent with previous findings (15). Our research confirmed that immunosuppression is a central mechanism in the pathophysiology of post-sepsis CCI. Notably, secondary infections were markedly more prevalent in the CCI group compared to the non-CCI group, with the most common types including urinary, respiratory, gastrointestinal, and bloodstream infections (particularly catheter-related infections). These infections serve as both a driving risk factor and a clinical hallmark of CCI, indicating their dual significance. This study provided further evidence that immunosuppression following pediatric sepsis contributes to the onset of secondary infections, which play a pivotal role in progression to CCI. Early detection and effective management of immunosuppression are crucial for preventing progression from pediatric sepsis to CCI and improving patient outcomes and long-term prognosis.

Multivariate logistic regression analysis identified several independent risk factors for the development of CCI in pediatric sepsis patients, including pSOFA score, respiratory system diseases, trauma, prolonged mechanical ventilation, surgical interventions, and secondary infections. Among these factors, prolonged mechanical ventilation demonstrated the highest predictive value, as evidenced by its largest AUC value. This strong predictive capacity may be linked to its association with critical illness myopathy (CIM), immunosuppression, and ICU-acquired weakness (ICU-AW). These complications increase the vulnerability of CCI patients to secondary infections, particularly ventilator-associated pneumonia, which can further prolong mechanical ventilation duration and exacerbate the risk of CCI. Thus, prolonged mechanical ventilation not only predicts the development of CCI but also plays a critical role in its pathogenesis. These findings highlight the importance of early recognition and targeted management of high-risk patients to mitigate the progression of sepsis to CCI. Immunosuppression, a central mechanism in this progression, significantly increases susceptibility to secondary infections, perpetuates organ dysfunction, delays recovery, and contributes to the chronic deterioration of the clinical condition. Understanding the role of immunosuppression in the development of CCI is crucial for advancing diagnostic and therapeutic strategies. Addressing this mechanism could lead to innovative treatment approaches aimed at improving the prognosis of sepsis patients. Furthermore, our study established immunosuppression as a hallmark of CCI, underscoring its pivotal role in shaping disease trajectory and outcomes.

This study has several limitations. As a single-center retrospective analysis, it is subject to potential selection bias, limiting the generalizability of its findings, and propose future multicenter prospective studies for validation. Additionally, the study included only a limited set of immune function evaluation markers, restricting the depth of immunological insights. The absence of dynamic follow-up data hindered the ability to track immunosuppression progression and its impact over time. Future research should explore the precise mechanisms underlying sepsis-induced immunosuppression and the development of strategies to modulate immune responses effectively. Such efforts are crucial to preventing and managing chronic critical illness, thereby reducing its incidence and improving long-term survival outcomes and quality of life for sepsis patients.

Our study indicated that CCI in pediatric sepsis was strongly linked to underlying respiratory diseases, trauma, elevated pSOFA scores, surgical procedures, and prolonged mechanical ventilation. These factors contributed to extended hospital stays, elevated secondary infection rates, and adverse clinical outcomes. The persistence of pro-inflammatory mediators and subsequent immunosuppression likely play a pivotal role in the progression to CCI within this population. By conducting a comprehensive analysis of the clinical features and risk factors related to CCI in pediatric sepsis, this study lays a crucial groundwork for the formulation of more targeted prevention and treatment approaches. This, in turn, holds the promise of substantially enhancing the outcomes for this susceptible patient group.

## Data Availability

The raw data supporting the conclusions of this article will be made available by the authors, without undue reservation.
